# Quercetin supplementation promotes recovery after exercise-induced muscle damage: a systematic review and meta-analysis of randomized controlled trials

**DOI:** 10.5114/biolsport.2023.121320

**Published:** 2022-11-18

**Authors:** Daniel Rojano-Ortega, José Peña-Amaro, Antonio J. Berral-Aguilar, Francisco J. Berral-de la Rosa

**Affiliations:** 1 CTS-595 Research Group, University Pablo de Olavide, Seville, Spain; 2 Department of Morphological and Socio-sanitary Sciences, Córdoba’s University, Spain

**Keywords:** Dietary supplements, Muscle damage, Muscle soreness, Inflammation, Oxidative stress

## Abstract

Quercetin (Q) is one of the most frequently consumed flavonoids in the human diet. The purpose of this systematic review and meta-analysis was to determine the effects of Q supplementation on muscle damage, muscle soreness and biochemical markers of inflammation, antioxidant capacity and oxidative stress after intense exercise. A literature search of SPORTDiscus, PubMed, Web of Science and Scopus was performed from inception to May 31, 2022. Forest plots were generated with fixed or random-effect models and standardized mean differences (SMD). Data extraction and quality assessment were performed independently by two authors. After application of the inclusion and exclusion criteria, 13 studies with a total of 249 sedentary to well-trained participants were included. For all studies there were some concerns about the risk of bias. All but one study used a supplementation dosage of 1000 mg/day. Q supplementation accelerated recovery of muscle function and significantly decreased muscle soreness 0/24 h after exercise (SMD: -1.33; p = 0.03), creatine kinase levels 24/48 h after exercise (SMD: -1.15; p = 0.02), and post-exercise oxidative stress (SMD: -0.92; p = 0.03). However, Q supplementation had no effect on IL-6 concentration. Q supplementation with a dose of 1000 mg/day for periods of more than seven days and a maximum of 12 weeks appears to be a safe and efficacious strategy to reduce muscle damage and muscle soreness, as well as to enhance recovery after intense exercise in sedentary to well-trained young men. Systematic review registration: PROSPERO CRD42021266801.

## INTRODUCTION

Phenolic compounds are abundant molecules in our diet that are synthesized exclusively by plants, and have aromatic rings with one or more attached hydroxyl groups [1]. They have received considerable attention because their dietary consumption has been linked to the prevention of chronic diseases, such as cancer, diabetes, and cardiovascular disease, due to their antioxidant and anti-inflammatory activities [1–3]. Flavonoids are an important group of phenolic compounds, consisting of two aromatic rings and an additional oxygenated heterocycle called the C ring [4]. Based on the hydroxylation type and the differences in the C ring, flavonoids can be subdivided into different subgroups, including anthocyanins, flavan-3-ols, flavones, flavanones, and flavonols [5, 6].

Flavonoids have been reported to have beneficial health effects related to their antioxidative, anti-inflammatory, anti-mutagenic, and anti-carcinogenic properties [7–9]. Quercetin (Q) is one of the most frequently consumed flavonoids in the human diet and exerts a broad range of health-promoting physiological effects in humans [10]. Important dietary sources of Q include apples, onions, berries, tomatoes, shallots, red grapes, leafy greens, and tea. The term Q should be used to describe the aglycone only (the molecule without an attached sugar); however, the name is frequently used to refer to any quercetin-type molecules [11].

The antioxidant effects associated with flavonoids are due to their free radical-scavenging abilities [8, 9]. A free radical is an unstable and highly reactive atom or molecule, due to the presence of unpaired electrons in its valence shell [12]. Free radicals are a product of cellular metabolism. Free radicals derived from oxygen are referred to as reactive oxygen species (ROS) and, at low or moderate levels, they exert beneficial effects in cells [13]. The human body naturally renders ROS inactive with the endogenous antioxidant defence system in conjunction with exogenous antioxidants consumed through a balanced diet. However, intense physical exercise increases the production of ROS, which may be highly damaging to cells [14], and may lead to an excess of ROS or oxidants over the capability of the cell to mount an effective antioxidant response. This imbalance is called oxidative stress [15].

Intense or unaccustomed physical exercise frequently causes exercise-induced muscle damage (EIMD), particularly after eccentric actions. It is characterized by a temporary loss of force production and delayed onset muscle soreness (DOMS), and usually has a negative impact on exercise performance [16, 17]. EIMD has been described as a two-phase process [17, 18]. The first phase involves structural damage to myofibers caused by the mechanical work performed by the muscles, and an increase in the production of ROS, contributing to oxidative stress. The second phase is an inflammatory response resulting from leukocyte infiltration into the damaged tissues, initiating tissue repair and adaptation [19]. However, it increases the production of ROS and can prolong inflammation, oxidative stress and cellular damage, which may delay the complete recovery of muscle function [14].

There is growing interest in the consumption of anti-inflammatory and antioxidant food supplements to reduce inflammation and enhance recovery after exercise [20]. Although some evidence suggests that antioxidant supplements may hinder the specific cellular adaptations to exercise [21], a negative impact of antioxidant supplementation on exercise training adaptation has not been reported with natural antioxidant foods [22]. Specifically, supplementation with foods containing a high concentration of phenolic compounds, such as tart cherry, pomegranate, or curcumin, has been widely used in sports to enhance performance, attenuate symptoms from EIMD, and accelerate recovery [20, 23–26].

A large body of scientific literature has investigated the effects of Q supplementation on exercise performance, based on its antioxidant and anti-inflammatory properties [27, 28]. However, the results are contradictory and two systematic reviews and meta-analyses on Q supplementation and endurance performance draw different conclusions [29, 30]. The effects of Q supplementation on recovery after EIMD have also been extensively studied, but the results appear, likewise, inconclusive. However, to the best of our knowledge, no systematic review or meta-analysis has been performed on this subject. Therefore, this systematic review and meta-analysis was designed to determine the effects of Q supplementation on muscle damage, muscle soreness and biochemical markers of inflammation, antioxidant capacity, and oxidative stress after intense exercise based on the existing literature.

## MATERIALS AND METHODS

### Search strategy

This systematic review and meta-analysis was designed according to the guidelines of the Preferred Reporting of Systematic Reviews and Meta-Analyses (PRISMA) statement [31]. The protocol was registered at PROSPERO, an international database of prospectively registered reviews in health and social care (CRD42021266801). Two independent authors (D.R.O. and A.J.B.R.) performed the literature search, study selection, and data extraction. Any disagreement was resolved by consensus.

**FIG. 1:**
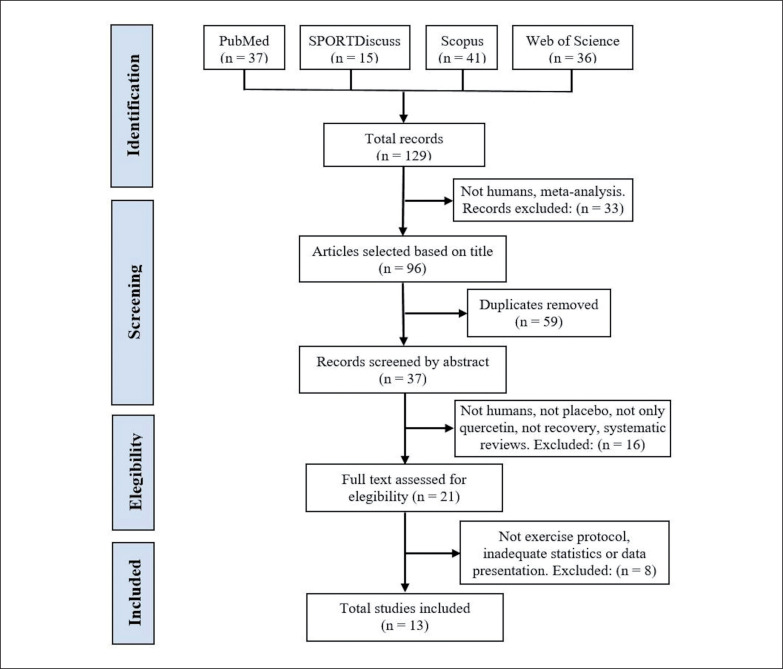
Prisma flowchart of the article selection process.

The literature search was conducted from inception to May 31, 2022, using four electronic databases: SPORTDiscus, PubMed, Web of Science and Scopus. The following search was performed: quercetin (Title) AND (muscle damage OR oxidative OR recovery OR muscle pain OR antioxidant OR inflammation OR soreness OR performance (Title)) AND (sports OR exercise OR train* OR athletes (Title/Abstract)). The search was limited to English language and journal articles. The search strategy is depicted in Figure 1.

### Inclusion and exclusion criteria

The studies included in this systematic review and meta-analysis met the following inclusion criteria: (i) research conducted with human participants, (ii) original articles in peer-reviewed publications, (iii) original studies that investigated the effect of Q supplementation intervention on muscle damage and recovery after a protocol to induce muscle damage, (iv) research conducted with one control/placebo (PLA) group, and (v) articles published in the period from inception to the end of May 2022. Exclusion criteria were: (i) research conducted with animals, (ii) non-English articles, (iii) studies that involved other interventions in addition to Q supplementation, (iv) systematic reviews or meta-analyses, and (v) studies that reported results inadequately or without adequate statistical analysis.

### Data extraction

The following data were extracted from each study: first author name, year of publication, intervention and placebo group characteristics, the dosage of supplements, supplementation duration, exercise protocol to induce muscle damage, and the effects of supplementation on functional measures, muscle soreness and biochemical markers of muscle damage, inflammation, antioxidant activity and oxidative stress.

### Quality assessment

The quality of the selected articles was assessed by two independent reviewers (D.R.O. and A.J.B.R.), using the Cochrane Collaboration risk of bias tool, which covers bias in six domains: selection bias, performance bias, detection bias, attrition bias, reporting bias, and other bias. Within each domain, assessments are made for one or more items, which may cover different aspects of the domain, or different outcomes [32]. Discrepant results were resolved through discussion.

### Statistical analysis

Review Manager software, version 5.4.1 (Cochrane Collaboration, Oxford, UK) was used to build the forest plot graphs and carry out the statistical analysis. The standardized mean difference (SMD) with a 95% confidence interval (CI) was used to present the results because units of measurements differed across studies. In the case of articles that reported the standard error of the mean (SEM), the standard deviation (SD) was calculated using the following formula: SD = SEM × sqrt (*n*), where *n* is the number of subjects. When necessary, the data were calculated from figures via WebPlotDigitizer. It is a program that converts graphical data to numerical data through manual plotting with high reliability [33] and has been used in other meta-analyses [34, 35].

Statistical heterogeneity of the treatment effects among studies were assessed using Cochran’s Q test and the inconsistency I^2^ test, and random-effect (I^2^ > 50%, P < 0.1) or fixed-effect models were used. A sensitivity analysis using the one-study removed method was also conducted to determine the influence of each study on the overall results. The calculated effect sizes (ES) were interpreted using the conventions outlined for SMD: < 0.2, trivial; 0.2–0.6, small; > 0.6–1.2, moderate; > 1.2–2.0, large; > 2.0–4.0, very large; > 4.0, extremely large [36].

### Publication bias

Potential publication bias was not evaluated because there were fewer than 10 studies included in each meta-analysis and this number is usually chosen as a threshold value to test publication bias [37].

## RESULTS

### Search results

The literature search provided a total of 129 articles identified through the combined descriptors. After examination of the titles, 33 articles were excluded because they were meta-analyses or they were not conducted in humans. After the elimination of duplicates, 37 articles were selected for abstract screening, of which 16 were excluded for not being conducted in humans, not having a PLA group, carrying out a supplementation other than solely Q, not studying recovery, or for being systematic reviews. Twenty-one studies were then selected for full-text reading, and 8 of these were excluded for not performing an exercise protocol to induce muscle damage, for reporting results inadequately, or for inadequate statistical analysis. The final number of studies included in this systematic review and meta-analysis was 13 [38–50]. A summary of the search process is depicted in Figure 1.

### Study characteristics

The characteristics of the included studies are summarized in Table 1. All selected studies were randomized placebo-controlled trials, except for that of Demirci [43], who did not mention whether participants were allocated at random. Six trials had a parallel-group design and the other 7 had a cross-over design. The total number of participants was 249 with sample sizes ranging from 10 to 20 participants in each group, except for Sholten and Sergeev [42], who only had 5 participants in the Q group and 3 in the PLA group. Only 5 studies [44, 46–48, 50] carried out an a priori statistical power analysis. Therefore, the other studies may not have used adequate sample sizes.

**TABLE 1:** Characteristics of the included studies.

Study	Participants	Groups	Age (years)	Quercetin content (mg/day)	Supplementation period	Exercise protocol to induce muscle damage
Nieman et al. [38]	Trained male cyclists	20 (Q) 20 (PLA)	26.1 ± 1.8 29.1 ± 2.4	1000 mg/day	24 days (exercise on days 22, 23 and 24)	3 h cycling bout at ~57% maximal work rate

McAnulty et al. [39]	Trained male cyclist	20 (Q) 20 (PLA)	26.1 ± 1.8 29.1 ± 2.4	1000 mg/day	24 days (exercise on days 22, 23 and 24)	3 h cycling bout at ~57% maximal work rate

Abbey & Rankin [40]	Healthy male team-sport-trained athletes	15 (Q) 15 (PLA)	23.3 ± 2.6 COD	1000 mg/day	7 days (exercise on day 8)	Repeated sprint test at maximum effort (12 × 30 m)

O’Fallon et al. [41]	Sedentary to recreationally active healthy males and females	8 MEN (Q) 7 WOM (Q) 7 MEN (PLA) 8 WOM (PLA)	19.5 ± 1.1 19.6 ± 1.3 20.9 ± 1.8 20.6 ± 1.1	1000 mg/day	14 days (exercise on day 9)	24 maximal eccentric contractions of EF

Sholten & Sergeev [42]	Male runners from a local club and collegiate cross country teams	5 (Q) 3 (PLA)	24.0 ± 5.4 22.5 ± 3.0	1000 mg/day	Six weeks (exercise on days 43–44)	10 km time trial treadmill run

Demirci [43]	Healthy licensed male boxers	10 (Q) 10 (PLA)	18.90 ± 1.10 19.00 ± 1.05	500 mg/day	30 days (exercise on day 31)	2 hours of normal boxing training the last supplementation day

Duranti et al. [44]	Healthy young men	14 (Q) 14 (PLA)	25.5 ± 0.8 COD	1000 mg/day	14 days (exercise on day 15)	6 to 10 sets of 10 maximal lengthening contractions of the EF

Gholami & Ardestani [45]	Moderately trained female adolescent swimmers	10 (Q) 10 (PLA)	15.30 ± 0.17 14.90 ± 0.12	1000 mg/day	14 days (exercise on day 15)	One minute bouts of crawl swimming (> 85% of maximum HR, 30 s-rest), until exhaustion

Patrizio et al. [46]	Healthy sedentary to recreationally men	10 (Q) 10 (PLA)	22.1 ± 1.8 COD	1000 mg/day	One single dose 3 h before exercise	3 sets of 8 repetitions at 80% 1RM of eight different exercises

Bazzucchi et al. [47]	Moderately active men	12 (Q) 12 (PLA)	26.1 ± 3.1 COD	1000 mg/day	14 days (exercise on day 15)	10 bouts (30 s-rest) of 10 maximal eccentric contractions of the EF.

Bazzucchi et al. [48]	Healthy low to moderately active men	16 (Q) 16 (PLA)	25.9 ± 3.3 COD	1000 mg/day	14 days (exercise on day 15)	10 bouts (30 s-rest) of 10 maximal eccentric contractions of the EF.

Sgrò et al. [49]	Healthy moderately active men	12 (Q) 12 (PLA)	25.67 ± 3.87 COD	1000 mg/day	14 days (exercise on day 15)	10 bouts (30 s-rest) of 10 maximal eccentric contractions of the EF.

Tsao et al. [50]	Healthy physically active men	12 (Q) 12 (PLA)	20.79 ± 0.53 COD	1000 mg/day	7 days (exercise on day 8)	Cycling test to exhaustion at 75% of VO_2max_

Q: quercetin group; PLA: placebo group; COD: cross-over design; EF: elbow flexors; HR: heart rate; RM: repetition maximum.

All selected studies were conducted on healthy subjects, 11 were conducted on men, 1 on women, and 1 on both women and men. The mean age of the participants ranged from 14.90 ± 0.12 to 29.1 ± 2.4 years. Nine studies evaluated the effects of Q supplementation on trained subjects, 3 on sedentary or low to moderately active subjects, and 1 did not mention the fitness level of participants. The protocol to induce muscle damage differed greatly across the included studies. One study evaluated the effects of a single dose of 1000 mg of Q taken 3 h before the protocol to induce muscle damage [46]. The remaining studies evaluated the effects of regular Q ingestion, for more than 7 days before exercise, with a dose of 1000 mg/day except for the study of Demirci [43], who used 500 mg/day.

### Methodological quality

**FIG. 2:**
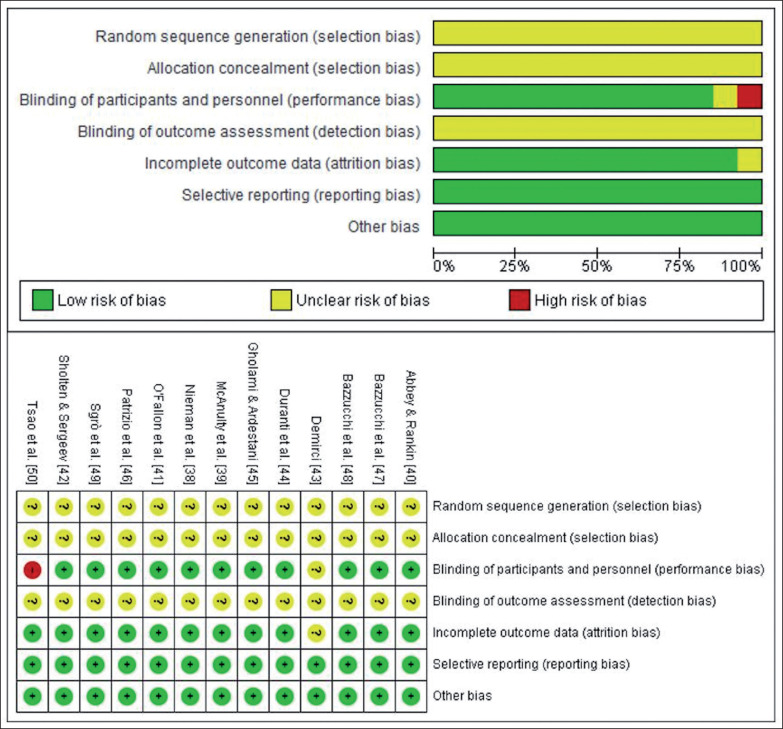
Methodological quality assessment of included trials.

Details of the methodological quality of the studies included are shown in Figure 2. The generation of the randomization sequence and the method of concealment were not described in any of the studies. Only one study [50] had a single blind design and one study [43] did not provide information for blinding of participants and personnel. None of the studies reported information about the blinding of the outcome assessment. Results were fully reported in all studies and there was no evidence of other bias in any of the studies.

### Effects of Q supplementation on functional measures and muscle soreness

**FIG. 3:**
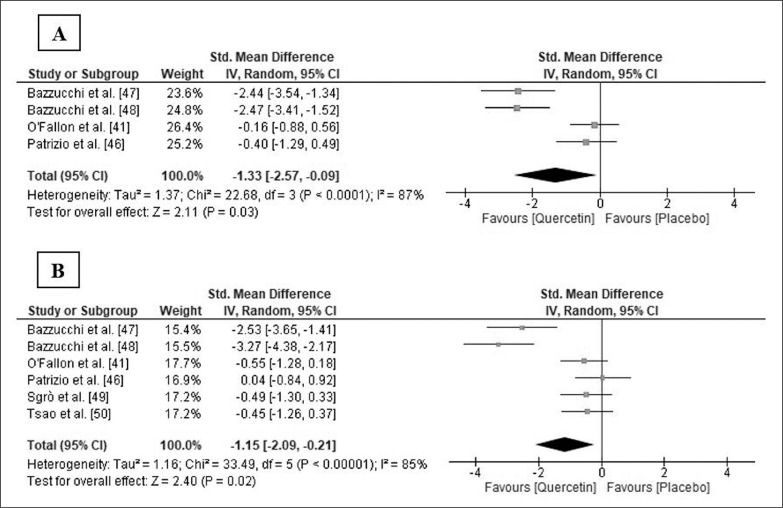
Forest plot showing the standardized mean differences and 95% confidence intervals for the effects of quercetin supplementation on muscle soreness (A) and creatine kinase (B).

Five studies [41, 46–49] measured one or more functional variables. Four studies [46–49] found a significant improvement in the Q group after supplementation in any of the variables measured after exercise or throughout the entire recovery period, when compared to the PLA group (Table 2). Four of those studies also measured muscle soreness [41, 46–48], but only Bazzucchi et al. [47] observed a trend of lower muscle soreness values after exercise in the Q group (Table 2). Meta-analysis indicated that soreness significantly decreased 0/24 h after exercise following Q supplementation (SMD: -1.33; 95% CI: [-2.57, -0.09]; p = 0.03; I^2^ = 87%; Figure 3a). The relative weight of each study in the analysis varied between 23.6% and 26.4%, demonstrating an equilibrated weight distribution. Although the results were not always significant, sensitivity analysis also showed a decrease in muscle soreness following Q supplementation.

**TABLE 2:** Variables measured and summary of findings of the included studies.

Study	Functional measures and muscle soreness	Biochemical markers of muscle damage, inflammation and oxidative stress	Significant differences between Q and PLA groups
Nieman et al. [38]		IL-1ra, IL-6, IL-8, IL-10, MCP-1, TNF-α. Measurements: each day pre and post.	↓ IL-8 and TNF-α post the three days (tendency: p < 0.1)

McAnulty et al. [39]	-----------------	CRP F2-IsoP, FRAP, TEAC Measurements: each day pre and post.	No significant differences

Abbey & Rankin [40]	-----------------	IL-6 XO, UA Measurements: baseline, pre, post and 1 h post.	No significant differences

O’Fallon et al. [41]	MIVC of EF, MVC of EF at 60 and 180º/s, soreness, REA. Measurements: pre, post (only MVC and REA), 24 h, 48 h, 72, 96 h and 120 h post.	CK IL-6, CRP Measurements: pre and 24 h, 48 h, 72 h, 96 h and 120 h post.	No significant differences

Sholten & Sergeev [42]	-------------	MDA, PC, TAC, SOD Measurements: pre and post.	↓ PC, SOD, TAC post Significant differences between groups not really measured

Demirci [43]	-------------	MDA, SOD, CAT, GSH Measurements: baseline and post.	↑ SOD, CAT, GSH ↓ MDA Significant differences between groups not really measured

Duranti et al. [44]	-------------	GSSH, GSH/GSSH, TBARS Measurements: baseline, pre and post.	↓ GSSG post ↑ GSH/GSSG post ↓ TBARS post

Gholami & Ardestani [45]	-------------	TNF-α Lactate Measurements: baseline, pre and post.	↓ TNF-α post

Patrizio et al. [46]	MVIC of the KE, MVC of KE at 30, 90, 180 and 240º/s, RTD200, soreness. Measurements: baseline, pre and post.	CK GSSH, GSH/GSSH Measurements: baseline, pre, post and 24 h post.	↑ MVC at 30, 90, 180 and 240 º/s post ↓ loss of MVIC of KE post ↑ RTD200 post

Bazzucchi et al. [47]	MVIC of EF, MVC of EF at 30, 60, 120 and 240 º/s, soreness, REA. Measurements: baseline, pre and post.	CK, LDH Measurements: baseline, pre, post. 24 h, 48 h and 72 h post.	↑ Relaxed EA post, ↑ MVIC pre and post ↑ MVC at 30 and 60 º/s post ↓ Soreness post (tendency) ↓ CK 48 and 72 h post ↓ LDH 24, 48 and 72 h post

Bazzucchi et al. [48]	MVIC of EF, MVC of EF at 30, 60, 120 and 240 º/s, soreness, REA. Measurements: baseline, pre, post, 24 h, 48 h, 72 h. 96 h and 7 days post.	CK, LDH Measurements: baseline, pre and 48 h, 72 h, 96 h and 7 days post.	↑ Relaxed EA post, ↑ MVIC post, 24 h, 72 h and 96 h post ↑ MVC most velocities and time-points ↓ CK 72 and 96 h post ↓ LDH 48, 72 and 96 h post

Sgrò et al. [49]	MVIC of EF Measurements: pre and 24, 48 h, 72 h, 96 h and 7 days post.	CK, LDH, MB IL-6 Measurements: pre and 24, 48 h, 72 h, 96 h and 7 days post.	↑ MVIC in all recovery time points ↓ CK 72 and 96 h post ↓ LDH in all recovery time points ↓ MB 48, 72 and 96 h post ↓ IL-6 48 and 72 h post

Tsao et al. [50]	-------------	CK, MB IL-6, TNF-α, CRP Measurements: pre, post, 1 h and 24 h post. SOD, MDA, TAC. Measurements: pre, post and 1 h post.	↓ CK 24 h post ↓ IL-6 post and 1 h post ↑ TAC 1 h post ↑ SOD pre, post and 1 h post ↓ MDA post

MIVC: maximal isometric voluntary contraction; EF: elbow flexors; MVC: maximal voluntary contraction; RAA: relaxed elbow angle; KE: knee extensors; RTD200: isometric rate of torque development the first 200 ms; IL: interleukin; MCP-1: monocyte chemoattractant protein 1; TNF-α: tumor necrosis factor alpha; CRP: C-reactive protein; F2-IsoP: F2-Isoprostanes; FRAP: ferric reducing ability; TEAC: trolox equivalent antioxidant capacity; XO: xanthine oxidase; UA: uric acid; CK: creatine kinase; MDA: malondyaldehyde; PC: protein carbonyls; TAC: total antioxidant capacity; SOD: superoxide dismutase; CAT: catalase; GSH: reduced glutathione; GSSG: oxidized glutathione; GSH/GSSH: reduced to oxidized glutathione ratio; TBARS: thiobarbituric acid reactive substances; LDH: lactate dehydrogenase; MB: myoglobin.

### Effects of Q supplementation on biochemical markers of muscle damage

Six studies [41, 46–50] analysed serum or plasma concentration of creatine kinase (CK), and three of them [47–49] also determined the plasma concentration of lactate dehydrogenase (LDH). Bazzucchi et al. [47, 48] and Sgrò et al. [49] found significantly lower plasma CK and LDH levels in the Q group at some point or throughout the entire recovery period after exercise, and Tsao et al. [50] also observed lower plasma CK levels in the Q group 24 h after exercise (Table 2). The pooled ES demonstrated that CK level significantly decreased in the Q group 24/48 h after exercise (SMD: -1.15; 95% CI: [-2.09, -0.21]; p = 0.02; I^2^ = 85%; Figure 3b). The relative weight of each study ranged from 15.4% to 17.7%, showing an equilibrated weight distribution. In the sensitivity analysis, the results remained consistent across all deletions.

### Effects of Q supplementation on markers of inflammation

Seven studies [38–41, 45, 49, 50] measured one or more markers of inflammation in plasma or serum. Gholami and Ardestani [45] found a significantly lower plasma concentration of tumour necrosis factor alpha (TNF-α) in the Q group after exercise, and Sgrò et al. [49] and Tsao et al. [50] observed lower plasma interleukin-6 (IL-6) levels at some point of the recovery period. The details of the effects of Q supplementation on markers of inflammation are summarized in Table 2.

**FIG. 4:**
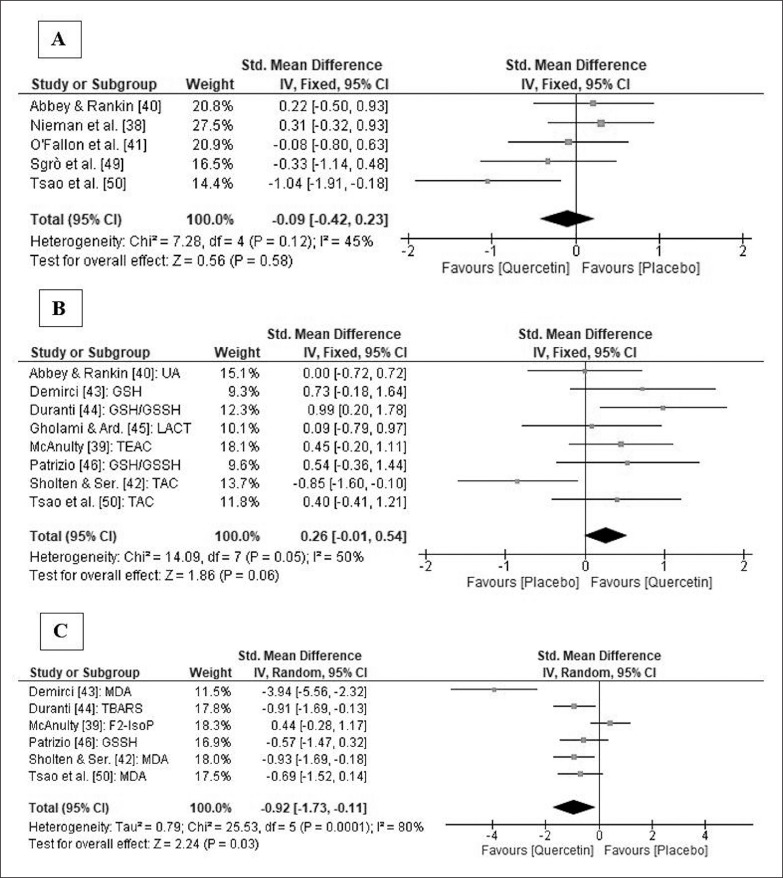
Forest plot showing the standardized mean differences and 95% confidence intervals for the effects of quercetin supplementation on IL-6 (A), markers of antioxidant capacity (B) and markers of oxidative stress (C). Note: IL-6, interleukin 6; UA, Uric Acid; GSH, reduced glutathione; GSH/GSSH, reduced to oxidized glutathione ratio; LACT, lactate; TEAC, trolox equivalent antioxidant capacity; TAC, total antioxidant capacity; MDA, malondyaldehyde; TBARS, thiobarbituric acid reactive substances; F2-IsoP, F2-isoprostanes; GSSH, oxidized glutathione.

A meta-analysis on IL-6 concentrations showed no significant differences between groups immediately after exercise (SMD: -0.09; 95% CI: [-0.42, 0.23]; p = 0.58; I^2^ = 45%; Figure 4a) and, in the sensitivity analysis, the results remained consistent across all deletions.

### Effects of Q supplementation on antioxidant activity and oxidative stress

Eight studies [39, 40, 42–46, 50] measured one or more markers of antioxidant activity in plasma or serum. Only three studies [43, 44, 50] found higher antioxidant capacity in the Q group compared to the PLA group (Table 2). Seven studies [39, 40, 42–44, 46, 50] measured one or more markers of oxidative stress in plasma or serum. Four of them [42–44, 50] observed lower levels of oxidative stress in the Q group than in the PLA group.

Two meta-analyses with different non-enzymatic markers of antioxidant capacity and oxidative stress were performed. Q supplementation produced a near significant increase in antioxidant activity after exercise (SMD: 0.26; 95% CI: [-0.02, 0.54]; p = 0.06; I^2^ = 50%). Following sensitivity analysis, the results remained unchanged across all deletions and became larger and significant when the study by Sholten and Sergeev was omitted [42]. Q supplementation also decreased oxidative stress (SMD: -0.92; 95% CI: [-1.73, 0.11]; p = 0.03; I^2^ = 80%). This ES was robust in the sensitivity analysis but became nearly significant (p = 0.06) after the omission of the studies by Demirci [43], Duranti et al. [44] and Sholten & Sergeev [42]. Forest plots presenting the impact of Q supplementation on antioxidant activity and oxidative stress, as well as the relative weight of each study, are shown in Figures 4b and 4c.

## DISCUSSION

Quercetin is one of the most frequently consumed flavonoids in the human diet and is widely distributed in fruits and vegetables. It has marked anti-inflammatory properties, as well as antioxidant activity, and exerts a protective effect against lipid peroxidation [51, 52]. Recent studies have investigated whether quercetin can reduce the extent of muscle damage and accelerate recovery after exercise in humans, but the results are not conclusive.

Consumption of Q is generally recognized as safe. According to Andres et al. [53], only mild adverse effects have been reported following high supplemental doses of isolated Q up to 1000 mg/day for a maximum duration of 12 weeks in adults. However, published data on the safety of long-term supplementation of more than 12 weeks with dosages of more than 1000 mg/day of isolated Q are currently not available, and long-term supplementation with a high dose of Q could have adverse effects in humans [53].

To the authors’ knowledge, this is the first systematic review and meta-analysis to examine the effectiveness of Q supplementation for recovery after EIMD in humans. Eleven studies met our inclusion criteria, involving a total of 249 participants. Our findings suggest that Q supplementation may accelerate recovery of muscle function and attenuate muscle damage and muscle soreness following strenuous exercise. Our results also suggest that Q supplementation is associated with reduced oxidative stress and increased antioxidant capacity. Therefore, Q supplementation is a good strategy to accelerate the recovery of muscle function.

### Effects of Q supplementation on functional measures and muscle soreness

Four of the five studies that analysed functional measures [41, 47–49] used supplementation for 14 days [41, 47, 48], whereas the remaining study used a single dose 3 hours before exercise [46]. Bazzucchi et al. [47, 48] observed lower strength loss of the elbow flexors and an increased relaxed elbow angle in the Q group after exercise; Patrizio et al. [46] and Sgrò et al. [49] also observed lower strength loss of the elbow flexors and the knee extensors, respectively. However, O’Fallon et al. [41] did not find significant differences between groups after exercise, probably because the exercise protocol, comprising 24 maximal eccentric contractions of the elbow flexors, was not sufficient to induce a large magnitude of muscle damage and, consequently, supplementation conferred no benefits for recovery. Therefore, it appears that Q supplementation is an effective strategy for recovery of functional measures after EIMD, even with a single dose before exercise, in sedentary to moderately active young athletes. These results may have been due to a higher neuromuscular efficiency after Q supplementation, because Q is an adenosine receptor antagonist, which improves nerve transmission and, consequently, increases muscular strength and reduces the perceived effort of exercise and fatigue [54, 55]. Our findings are broadly in agreement with those obtained in other systematic reviews on nutritional supplements [20, 25, 56].

Four studies measured muscle soreness with a 10-cm visual analogue scale (VAS). Q supplementation produced a significant reduction of muscle soreness 24/48 h after exercise in sedentary to moderately active young men (ES = -1.33, p = 0.03). The ES can be compared with meta-analyses conducted on other ergogenic aids. Rhim et al. [35] reported that supplementation with citrulline significantly reduced feelings of muscle soreness 24 h after exercise, with an ES of -0.99, and no significant association was detected 48 h after exercise between citrulline ingestion and muscle soreness. Lv et al. [57] found that supplementation with omega-3 polyunsaturated fatty acid significantly reduced muscle soreness 48 h days after eccentric exercise, with an ES of -0.93. Finally, Jones et al. [58] reported that nitrate-rich beetroot juice significantly reduced muscle soreness 48 h after exercise, with an ES of -0.56. Therefore, Q may be more effective for reducing muscle soreness than other nutritional supplements, because of its analgesic effect, which is dependent on many mechanisms including nitric oxide production, activation of γ-aminobutyric acid and serotonin receptors [59], and inhibition of pro-nociceptive cytokine production and oxidative stress [60].

### Effects of Q supplementation on markers of muscle damage

The three studies that analysed plasma LDH [47–49] found significantly lower levels in the Q group at some point or throughout the entire recovery period after exercise. In addition, the meta-analysis indicated that Q supplementation decreased CK levels 24/48 h after exercise in sedentary to physically active young men (ES = -1.15, p = 0.02). Comparisons with other meta-analyses show that Q supplementation appears to be more effective for reducing CK levels than other nutritional supplements. Jones et al. [58] noted no significant differences between the nitrate-rich beetroot juice group and the PLA 24 or 48 h after exercise, whereas Northeast and Clifford [61] observed significant differences in favour of the creatine supplementation group 48 h after exercise but with a moderate ES of -1.06. Our results may be explained by the role of Q in protecting muscle fibres from damage so that the extent of cell membrane disruption is lower, which attenuates the increase of CK [47]. However, the precise mechanism of this protective effect is still unclear.

### Effects of Q supplementation on markers of inflammation

The meta-analysis on IL-6 levels showed no differences between the Q and PLA groups after exercise. Our results are in line with the meta-analysis of nitrate-rich beetroot juice supplementation conducted by Jones et al. [58], who reported no significant differences in IL-6, IL-8, or TNF-a between the experimental and placebo groups after exercise. Likewise, even though Hill et al. [62] observed that tart cherry supplementation significantly decreased IL-6 after exercise, the overall ES was small.

### Effects of Q supplementation on antioxidant activity and oxidative stress

Q supplementation increased antioxidant activity after exercise in sedentary to trained young male and female athletes (ES = 0.26, p = 0.06), but the ES was small and only became significant when the study by Sholten and Sergeev [42] was removed (ES = 0.44, p = 0.004). Those authors explained that their unexpected result might have been due to subjects being randomized by maximal oxygen uptake rather than antioxidant activity, but it could also have simply been due to biological variations because the number of subjects was too low (5 in the Q group and 3 in the PLA group). The meta-analysis revealed that Q supplementation also decreased oxidative stress markers (ES = -0.92, p = 0.03). Most of the systematic reviews investigating the effects of supplementation with other ergogenic aids on recovery after EIMD did not include antioxidant activity or oxidative stress markers in their meta-analyses. Thus, we can only compare our ES with the results of Stepanyan et al. [63], but they did not observe significant protection of vitamin E against exercise-induced lipid peroxidation. Conversely, our results suggest that Q supplementation is effective for reducing oxidative stress and, to some extent, increasing antioxidant capacity. This is potentially due to the fact that Q is the most potent scavenger of ROS within the flavonoid family [64] because it has an optimal chemical structure for free radical scavenging, particularly because of the presence and location of the hydroxyl substitutions and the catechol group in the Bring [65, 66]. In addition, Q increases the body’s antioxidant capacity by regulating the levels of glutathione, which is necessary for the enzyme SOD to catalyse the decomposition of hydrogen peroxide to non-toxic H_2_O [67]. Despite Q supplementation acting directly in the secondary damage process via its antioxidant potential, it is possible to speculate that Q supplementation before exercise can reduce ROS production and limit its potentially harmful effect on cells, increasing the cell membrane resistance to the same mechanical stress [68] and therefore reducing the amount of damage caused by exercise.

### Other considerations and practical applications

The results of the study by Sgrò et al. [49] suggest a new possible reason for Q to modulate damage and recovery. It is known that insulin-like growth factors 1 (IGF-I) and 2 (IGF-II) are produced during the recovery period after EIMD and both factors play an important role in skeletal muscle regeneration and remodelling [69, 70]. The authors analysed the influence of Q supplementation on circulating levels of anabolic IGF-I and IGF-2, and they concluded that Q supplementation is a good strategy to promote recovery after EIMD because it increases plasma levels of anabolic factors IGF-I and IGF-II [49]. New studies are needed to support their conclusion.

Some practical applications can be drawn from this study: 1) Regular quercetin supplementation is a good strategy to reduce muscle damage and muscle soreness caused by strenuous exercise. 2) Regular quercetin supplementation also leads to a reduction of oxidative stress and increases, to some extent, antioxidant capacity. 3) One week of quercetin supplementation in doses of 1000 mg/day seems to be enough to obtain positive results.

### Study limitations

There are several limitations to the current study. First, many studies did not report the necessary data for the meta-analyses. Second, the exercise protocol varied substantially between studies, inducing different levels of muscle damage. Moreover, the different training statuses of the participants and the size of the muscles involved affected the magnitude of the muscle damage experienced. Third, most studies only included young male participants; hence, these findings are unlikely to be generalizable to females or older adults. Fourth, most studies did not perform a power analysis, and the small sample sizes were probably insufficient to detect between-group differences. In addition, though the quality of the included studies was generally acceptable, the randomization, allocation concealment, and blinding of outcome assessment procedures were inadequately described. Lastly, it was necessary to use different parameters to conduct the meta-analyses regarding antioxidant activity and oxidative stress. Although this procedure is not uncommon in the existing literature, it must be kept in mind when interpreting the results.

## CONCLUSIONS

The results of our systematic review and meta-analysis support the effectiveness of Q supplementation for reducing muscle damage and accelerating recovery after intense exercise in young male athletes. However, a limited number of studies were included in the different meta-analyses and the ES were not significant for some variables except when certain studies were removed. Therefore, our findings should be interpreted cautiously. Despite these limitations, based on the studies included in this investigation and the potential risks discussed by Andres et al. [53], Q supplementation with a dose of 1000 mg/day for periods of more than seven days and a maximum of 12 weeks appears to be a safe and efficacious strategy to reduce muscle damage and muscle soreness, as well as to enhance recovery in sedentary to well-trained young men. Athletes may also benefit from ingesting a single dose before exercise, but only one study investigated acute supplementation, and further research is needed. More evidence is also required to confirm the efficacy of Q supplementation for females and older adults.
